# The use of noncrystallographic symmetry averaging to solve structures from data affected by perfect hemihedral twinning

**DOI:** 10.1107/S2053230X16000923

**Published:** 2016-02-16

**Authors:** Charles Sabin, Pavel Plevka

**Affiliations:** a Central European Institute of Technology – Masaryk University, Kamenice 653/25, 625 00 Brno, Czech Republic

**Keywords:** hemihedral perfect twinning, noncrystallographic symmetry averaging, molecular replacement, virus structure, symmetry, detwinning, twin domain, mero­hedral twinning, mask envelope

## Abstract

Hemihedral twinning is a crystal-growth anomaly in which a specimen is composed of two crystal domains that coincide with each other in three dimensions. However, the orientations of the crystal lattices in the two domains differ in a specific way. In diffraction data collected from hemihedrally twinned crystals, each observed intensity contains contributions from both of the domains. With perfect hemihedral twinning, the two domains have the same volumes and the observed intensities do not contain sufficient information to detwin the data. Here, the use of molecular replacement and of noncrystallographic symmetry (NCS) averaging to detwin a 2.1 Å resolution data set for *Aichi virus 1* affected by perfect hemihedral twinning is described. The NCS averaging enabled the correction of errors in the detwinning introduced by the differences between the molecular-replacement model and the crystallized structure. The procedure permitted the structure to be determined from a molecular-replacement model that had 16% sequence identity and a 1.6 Å r.m.s.d. for C^α^ atoms in comparison to the crystallized structure. The same approach could be used to solve other data sets affected by perfect hemihedral twinning from crystals with NCS.

## Abstract 2

Molecular replacement and noncrystallographic symmetry averaging were used to detwin a data set affected by perfect hemihedral twinning. The noncrystallographic symmetry averaging of the electron-density map corrected errors in the detwinning introduced by the differences between the molecular-replacement model and the crystallized structure.

## 1. Introduction   

*Aichi virus 1* (AiV1) is a member of the *Kobuvirus* genus belonging to the *Picornaviridae* family of small non-enveloped viruses (Yamashita *et al.*, 1991 ▸). The outer diameter of the virion is about 300 Å. The capsid of AiV1 is composed of 60 copies of each of the three capsid proteins VP0, VP1 and VP3 organized with icosahedral symmetry. The AiV1 genome is a single-stranded positive-sense RNA 8251 nucleotides in length (Yamashita *et al.*, 1998 ▸). Human infection by AiV1 can result in gastroenteritis (Yamashita *et al.*, 1998 ▸).

Twinning is a crystal-growth anomaly in which a crystal specimen is composed of domains whose orientations give rise to overlapping diffraction patterns (Redinbo & Yeates, 1993 ▸; Yeates & Fam, 1999 ▸; Chandra *et al.*, 1999 ▸; Helliwell, 2008 ▸; Grainger, 1969 ▸). In hemihedral twinning, the specimen is composed of two domains whose crystal lattices coincide with each other in three dimensions. Since the real-space lattices of the two domains coincide, the reciprocal lattices of the domains lie on top of each other (Yeates, 1997 ▸; Parsons, 2003 ▸). The domain sizes in the twinned crystals are presumed to be large compared with the coherence length of the X-ray beam, so the waves scattered from the separate domains do not interfere. Thus, in hemihedral twinning, each observed intensity *I*_obs_(**h**) is a weighted sum of the intensities of the reflections from the two domains *I*(**h**_1_) and *I*(**h**_2_), 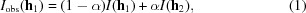


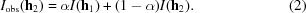



The twinning fraction (α) represents the part of the volume of the specimen occupied by the domain in the arbitrarily selected ‘first’ orientation. The domain in the ‘second’ orientation occupies the remaining (1 − α) part of the specimen volume. The case of α = 0 corresponds to an untwinned specimen. Cases where 0 < α << 0.5 are referred to as ‘partial twinning’ and cases where α approaches 0.5 as ‘perfect twinning’ (Parsons, 2003 ▸; Yeates, 1997 ▸). The two domains are related by the twinning operator, but not by their crystallo­graphic symmetry (Yeates, 1997 ▸; Parsons, 2003 ▸).

Partial hemihedral twinning does not obscure the true crystallographic symmetry because the pairs of reflections related by the twinning operator have different intensities (Parsons, 2003 ▸). A statistical analysis of the observed intensities can be used to estimate the twinning fraction α. The true crystallographic intensities can be calculated, using the α value, based on (3) and (4) (Grainger, 1969 ▸; Yeates, 1997 ▸):
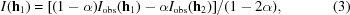


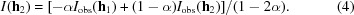



As the twinning fraction α approaches 0.5 the term (1 − 2α) approaches zero, and the true crystallographic intensities cannot be accurately calculated based on (3) and (4). With a perfect twin (twin fraction α = 0.5) the two reflections related by the twin law contribute equally to both of the observed intensities related by the twinning operator: *I*_obs_(**h**_1_) = 0.5*I*(**h**_1_) + 0.5*I*(**h**_2_) and *I*_obs_(**h**_2_) = 0.5*I*(**h**_1_) + 0.5*I*(**h**_2_) (Yeates, 1997 ▸). Therefore, the symmetry of the twinning operation is superimposed on top of the actual Laue symmetry and the apparent Laue group is of a higher order than the actual Laue group (Supplementary Fig. S1).

Here, we report the structure determination of an AiV1 virion based on diffraction data affected by perfect hemihedral twinning. The structure of AiV1 and its implications for the infection process and the design of antiviral compounds will be described elsewhere. This paper focuses on the utilization of noncrystallographic symmetry (NCS) averaging to solve a structure from a data set affected by perfect hemihedral twinning.

## 2. Materials and methods   

### 2.1. Virus growth and purification   

Green monkey kidney (GMK) cells were grown on 150 mm diameter plates to 70% confluency in Dulbecco’s Modified Eagle’s Medium (DMEM) supplemented with 10% foetal bovine serum (FBS; Sigma–Aldrich). 60 plates of green monkey kidney cells were infected with AiV1 with a multiplicity of infection (MOI) of 0.1 (GenBank AB040749.1; obtained from Dr A. Michael Lindberg, Linnaeus University, Sweden) and incubated at 37°C and 5% CO_2_ until the cytopathic effect was observed. Both cells and virus-containing supernatant were harvested. The cells were pelleted by centrifugation at 4500 rev min^−1^ at 4°C for 15 min in a Beckman JA-10 rotor and lysed by freezing and thawing three times followed by Dounce homogenization. The cell debris was removed by centrifugation at 10 000 rev min^−1^ for 15 min in a Beckman Coulter JA-10 rotor. The virus-containing supernatant was pooled with the previously harvested virus-containing tissue-culture medium. Polyethylene glycol 8000 and NaCl were added to final concentrations of 5% and 0.5 *M*, respectively. The virus was precipitated by overnight incubation at 4°C with gentle shaking. The precipitate was pelleted by 15 min centrifugation at 9500 rev min^−1^ and 4°C using a Beckman Coulter JA-10 rotor. The pellet was resuspended in buffer *A* [0.25 *M* 4-(2-hydroxyethyl)-1-piperazineethane­sulfonic acid (HEPES) pH 7.5, 0.25 *M* NaCl] and treated with MgCl_2_ (final concentration 0.005 *M*), DNase (final concentration 10 µg ml^−1^) and RNase (final concentration 10 µg ml^−1^) for 30 min at room temperature followed by trypsin (final concentration 80 µg ml^−1^) digestion for 10 min at 37°C. Subsequently, ethylendiaminetetraacetic acid and Nonidet P-40 were added to final concentrations of 0.015 *M* and 1%, respectively. The solution was centrifuged at 4500 rev min^−1^ for 10 min in a Beckman Coulter JA-10 rotor and the resulting pellet was discarded. The clarified supernatant was layered over a 30%(*w*/*v*) sucrose cushion in buffer *A* and centrifuged in a Beckman Coulter 50.2 Ti rotor at 48 000 rev min^−1^ for 2 h at 10°C. After centrifugation, the supernatant was discarded and the pellet was resuspended in 1 ml buffer *A*. The virus suspension was layered onto a 10–40% tartrate gradient and centrifuged in a Beckman Coulter SW 41 Ti rotor for 90 min at 36 000 rev min^−1^ and 4°C. The virus band was collected using a syringe with a needle. The virus was transferred to buffer *A* using repeated concentration and dilution steps of the virus solution in centrifugal concentrators with a 100 kDa cutoff membrane. The concentration of the purified virus was measured in a spectrophotometer using an absorption coefficient of 7.25 mg ml^−1^ cm^−1^ at 260 nm.

### 2.2. Crystallization and data collection   

The AiV1 crystals were grown using the hanging-drop vapour-diffusion method at 20°C with a well solution consisting of 0.05 *M* cadmium sulfate, 0.1 *M* HEPES pH 7.5, 1.0 *M* sodium acetate. Crystallization drops were prepared by mixing 1 µl well solution with an equal volume of the virus at a concentration of 3 mg ml^−1^ in buffer *A*. The crystals formed within one month. For diffraction experiments, the crystals were directly vitrified in liquid nitrogen without presoaking in any cryoprotectant. A single-crystal diffraction data set was collected using a Pilatus3 6M 100 Hz detector on beamline I03 at Diamond Light Source, UK. An oscillation range of 0.1° and a wavelength of 0.976 Å were used for data collection. The diffraction pattern extended to a resolution of 2.1 Å. The diffraction images were processed and scaled using *XDS* and *AIMLESS* from the *CCP*4 software package (Kabsch, 2010 ▸; Winn *et al.*, 2011 ▸).

### 2.3. Data deposition   

The model of AiV1 together with the observed structure-factor amplitudes and intensities was deposited in the Protein Data Bank as entry 5aoo. Detwinned structure-factor amplitudes and phases calculated from the refined model and refined by 30 cycles of NCS averaging were also deposited.

## 3. Results and discussion   

### 3.1. Detection of twinning in the AiV1 diffraction data   

**Figure 1:**
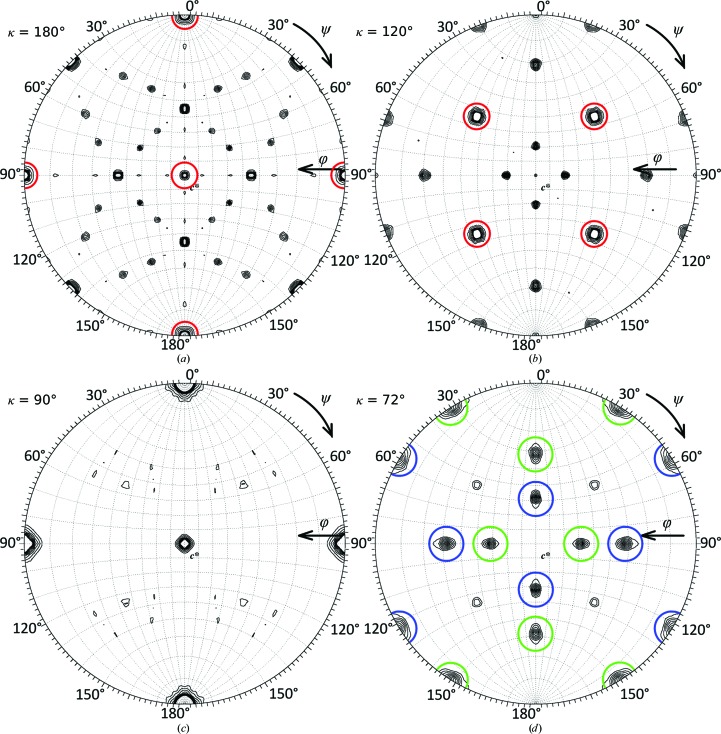
Sections of the rotation function calculated from AiV1 diffraction data processed in space group *I*23. Stereographic plots of (*a*) κ = 180°, (*b*) κ = 120°, (*c*) κ = 90° and (*d*) κ = 72° rotation-function sections were calculated using 12–7 Å resolution AiV1 diffraction data and a 150 Å radius of integration. The plots were contoured in 0.5σ increments of the rotation-function values starting from 1.0σ. Peaks of shared crystallographic and icosahedral symmetry are highlighted with red circles in (*a*) and (*b*). All of the remaining non-noise peaks belong to NCS symmetry. Fivefold symmetry peaks corresponding to the twin domains are differentiated by blue and green circles in (*d*).

The AiV1 crystal diffracted to a resolution of 2.1 Å. The diffraction pattern was compatible with a body-centred lattice and had 432 point symmetry (Table 1 ▸; Evans, 2006 ▸). Since there was no indication of systematic absences along the fourfold axis (reflections *h*00: *h* = 4*n*) the space group was identified as *I*432. The presence of the crystallographic and NCS axes belonging to the symmetry of the crystallized AiV1 virions is shown in the rotation-function sections for twofold, threefold, fourfold and fivefold symmetry (Fig. 1 ▸; Tong & Rossmann, 1990 ▸). Statistical analysis did not indicate twinning (Table 2 ▸; Evans, 2006 ▸; Padilla & Yeates, 2003 ▸). However, no molecular-replacement solution with good packing was obtainable in space group *I*432 (Supplementary Fig. 2*a*). The unit-cell volume and the rotation-function plots indicated that a crystal with the same unit-cell parameters but with *I*23 symmetry (a subset of *I*432 symmetry) would allow the packing of AiV1 virions (Supplementary Fig. S2*b*). This provided evidence that the crystal may be hemihedrally twinned, with the two domains composed of *I*23 unit cells rotated 90° relative to each other. Since the fourfold symmetry axes owing to the twinning had a similar *R*_meas_ as the twofold and threefold axes belonging to the *I*23 crystallographic symmetry (Table 1 ▸) the data were perfectly hemihedrally twinned.

### 3.2. Data processing   

For the twinning analyses and the calculation of rotation functions, the AiV1 diffraction images were processed in space group *I*23 to a resolution of 2.3 Å using *XDS* (Kabsch, 2010 ▸). However, after determining that the diffraction data were affected by perfect hemihedral twinning, the diffraction images were reprocessed in space group *I*432. Taking advantage of the higher symmetry enabled us to obtain a data set that was more than 90% complete from the first 160 diffraction images (total rotation range of 16°). The data could be processed to a resolution of 2.1 Å (Table 3 ▸). Subsequently, the diffraction data were expanded from *I*432 to *I*23 using *SFTOOLS* from *CCP*4 (Winn *et al.*, 2011 ▸).

### 3.3. Molecular-replacement solution   

The twofold and threefold rotation-function plots indicated that a subset of the icosahedral twofold and threefold symmetry axes were aligned with the crystallographic cubic symmetry axes (Figs. 1 ▸ *a* and 1 ▸ *b* and Supplementary Fig. S2*b*). Icosahedral 532 symmetry elements can be aligned with those of 23 symmetry in two equivalent choices that are rotated 90° relative to one another. However, both of the icosahedral orientations were present because the two crystal twin domains are related by a 90° rotation (Figs. 1 ▸ *a*, 1 ▸ *b* and 1 ▸ *d* and Supplementary Fig. S2*b*). To verify the consistency of the particle in the standard orientation with the experimental data, values of the icosahedral locked rotation function with the twofold icosahedral axes aligned with the coordinate axes were calculated for symmetry rotated 0 and 90° about the *z* axis using the data processed in space group *I*23. Similar values of the locked rotation function at rotations of 0 and 90° (4.7 and 4.3σ, respectively) verified that the data were affected by perfect hemihedral twinning. Superimposition of the icosahedral symmetry with the cubic 23 symmetry resulted in the crystallographic asymmetric unit containing five icosa­hedral asymmetric units (1/12 of a virus particle).

Based on the orientation of the icosahedral particle and the crystal-packing considerations, the virus particle had to be positioned with its centre at the origin of the unit cell. Because of the body centring, there is another virus particle in the centre of the unit cell (Supplementary Fig. S2*b*).

### 3.4. Detwinning of the measured intensities based on the molecular-replacement model and NCS averaging   

Detwinning of the diffraction data collected from crystals affected by perfect hemihedral twinning (α = 0.5) cannot be based on comparisons of the observed reflection intensities (see equations 3 and 4; Yeates & Fam, 1999 ▸; Yeates, 1997 ▸). However, a simulated twinned data set can be calculated if a model of the structure is available by summing the intensities of the reflections derived from the model with the intensities of the reflections related by the twinning operator. The ratio between the two twin-related intensities can be used to detwin the corresponding measured intensities. Thus, a known structure can supply the information necessary to detwin data affected by perfect hemihedral twinning. The detwinning procedure was performed using the following steps.

In a preparation step, PDB models of ten picornaviruses (PDB entries 1bev, 1cov, 1ev1, 1hxs, 1tmf, 2mev, 2wff, 2x5i, 3vbf and 4iv1) were positioned in the unit cell according to the molecular-replacement solution described above (Muckel­bauer *et al.*, 1995 ▸; Smyth *et al.*, 1995 ▸; Filman *et al.*, 1998 ▸; Miller *et al.*, 2001 ▸; Luo *et al.*, 1992 ▸; Krishnaswamy & Rossmann, 1990 ▸; Tuthill *et al.*, 2009 ▸; Plevka *et al.*, 2010 ▸; Wang *et al.*, 2012 ▸; Porta *et al.*, 2013 ▸). *CNS* was used to calculate the phases and structure-factor amplitudes based on the models and the five NCS operators defining the relative positions of the icosahedral asymmetric units in the crystallographic asymmetric unit (Brunger, 2007 ▸). The best molecular-replacement solution was obtained using the 1cov structure (Table 4 ▸). The resulting *CNS* reflection file was converted to MTZ format using *F*2*MTZ* from *CCP*4 (Winn *et al.*, 2011 ▸). This procedure provided the initial model-derived structure-factor amplitudes and phases.

The following procedure was then used iteratively.

(i) The twin symmetry (*k*, *h*, −*l*) was used to generate a version of the calculated structure-factor amplitudes rotated 180° around the [110] axis, corresponding to the second twin domain, using *REINDEX* from *CCP*4. The reflections were sorted according to the *CCP*4 *h*, *k*, *l* convention using *CAD* (Winn *et al.*, 2011 ▸).
(ii) The calculated structure-factor amplitudes of both of the twin domains were squared to obtain estimates of the reflection intensities. A twinning ‘portion’ was calculated for each reflection based on (5).
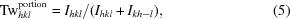
Please note that each reflection had a different twinning portion. This is in contrast to the twinning fraction α, discussed above, that characterizes the ratio of the twin domains, which is the same for all reflections from a particular data set affected by hemihedral twinning. The detwinned intensity of each reflection was calculated by multiplying the observed intensity value by the corresponding twinning portion (6). The detwinning was performed using *SFTOOLS* (Winn *et al.*, 2011 ▸).
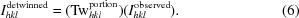

(iii) The detwinned intensities were converted to structure-factor amplitudes using *TRUNCATE* (Winn *et al.*, 2011 ▸).
(iv) The phases and the structure-factor amplitudes calculated from the model were combined with the detwinned structure-factor amplitudes using *SFTOOLS*. The structure-factor amplitudes calculated from the model were scaled to the detwinned structure-factor amplitudes using *RSTATS* (Winn *et al.*, 2011 ▸). *RSTATS* also produced a scaling *R* factor and correlation coefficient comparing the scaled |*F*_calc_| and |*F*_obs_^detwinned^|, which enabled the agreement between the observed and the model-derived data to be monitored.
(v) An electron-density map (2|*F*_obs_^detwinned^| − |*F*_calc_|), φ_calc_ was calculated using *FFT* (Winn *et al.*, 2011 ▸).
(vi) The electron-density map was averaged according to the fivefold NCS using *AVE* from the Uppsala Software Factory package (Kleywegt & Read, 1997 ▸). Two masks were used sequentially for the electron-density averaging. An initial mask was calculated based on the structure of *Bovine enterovirus* (BEV; PDB entry 1bev) by including all voxels within 5 Å of any atom of the model using *MAMA* (Kleywegt & Jones, 1999 ▸; Smyth *et al.*, 1995 ▸). After ten cycles, the mask derived from the BEV model was replaced with a correlation map-based mask (see below for a description of the preparation of the correlation map-based mask).
(vii) Improved structure-factor amplitudes and phases were calculated from the averaged map using *SFALL* (Winn *et al.*, 2011 ▸).
(viii) The procedure was cyclically repeated 30 times from step (i).

### 3.5. Model bias introduced by the detwinning procedure and its mitigation by NCS map averaging   

**Figure 2:**
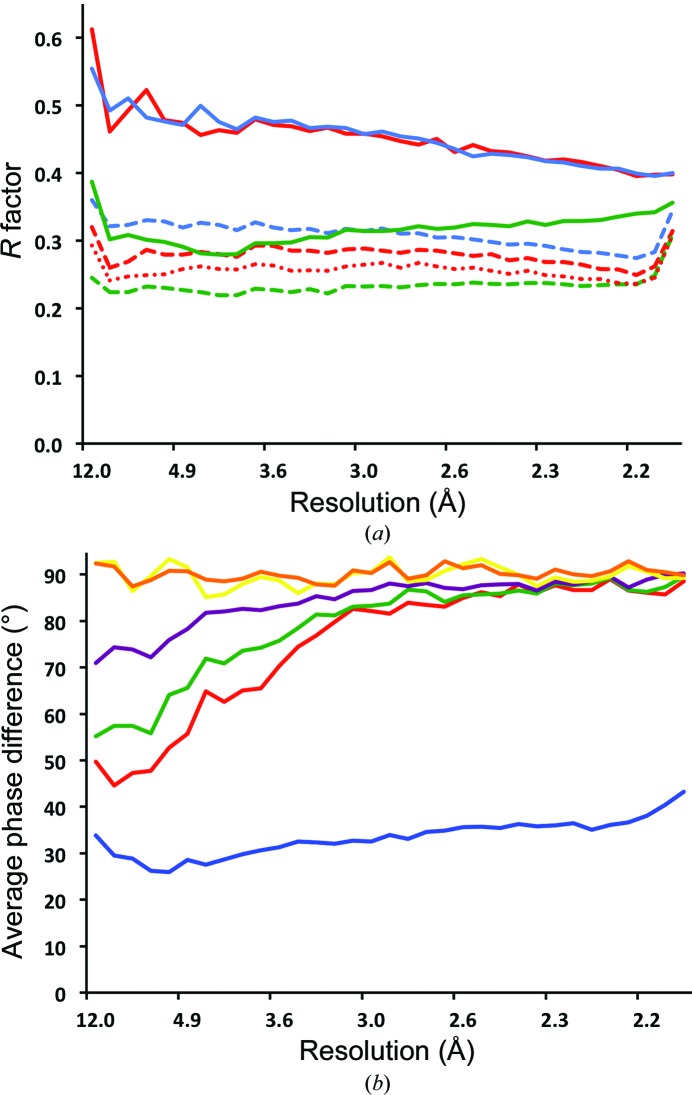
(*a*) Crystallographic *R* factors comparing |*F*_obs_^detwinned^| and |*F*_calc_| as a function of resolution at different stages of AiV1 structure determination. The *R* factor comparing |*F*_obs_^detwinned^| and |*F*_calc_| calculated from the BEV model converted to polyalanine is shown as a continuous red line, the *R* factor after refinement by ten cycles of NCS averaging using the BEV-derived mask is shown as a dashed red line and the *R* factor after 30 cycles of NCS averaging using the correlation map-based mask is shown as a dotted red line. The *R* factor comparing |*F*_obs_^detwinned^| and |*F*_calc_| calculated from the final AiV1 model is shown as a continuous green line and the *R* factor after ten cycles of NCS averaging as a dashed green line. The *R* factor comparing |*F*_obs_^detwinned^| and |*F*_calc_| calculated from the φCb5 structure is shown as a continuous blue line and the *R* factor after ten cycles of NCS averaging is shown as a dashed blue line. (*b*) Phase-difference plots comparing phases at various stages of structure determination with phases derived from the final AiV1 structure and refined by 30 cycles of NCS averaging. Phase differences were calculated in narrow resolution bins and plotted against resolution. The average phase difference of phases of the BEV model are shown as a violet line, of the BEV model refined by ten cycles of the NCS averaging using the BEV-derived averaging mask as a green line, of the BEV model refined by 30 cycles of the NCS averaging using the correlation-map based mask as a red line, of the φCb5 model as an orange line, of the φCb5 model refined by ten cycles of the NCS averaging as a yellow line and of the final AiV1 structure as a blue line. (See text for further details.)

An electron-density map calculated using the detwinned structure-factor amplitudes (2|*F*_obs_^detwinned^| − |*F*_calc_|) and phases φ_calc_ is affected by more extensive model bias than is present in the map calculated from data that are not twinned (2|*F*_obs_| − |*F*_calc_|), φ_calc_. The additional model bias is owing to the application of the twinning portions that are derived from the molecular-replacement model (equations 5 and 6). The differences between the molecular-replacement model and the actual crystallized structure result in errors in the twinning portions that subsequently introduce errors into the detwinned amplitudes |*F*_obs_^detwinned^|. Thus, the detwinning procedure limits the amount of information in the (2|*F*_obs_^detwinned^| − |*F*_calc_|), φ_calc_ map calculation that is provided by the |*F*_obs_| values. In addition, as in the standard molecular-replacement map calculation, the differences between the model and the crystallized structure result in errors in phases (φ_calc_) that introduce model bias. However, the NCS averaging effectively increases the observed data redundancy, since volumes of the NCS-related molecules are forced to have the same electron-density distributions. Differences between the NCS-related positions owing to errors in the twinning portions and phases are removed by the NCS averaging. The resulting averaged electron-density map can be used to calculate improved phases and twinning portions that better resemble the crystallized structure. The averaging procedure combined with detwinning resulted in an improvement in the *R*-factor value on comparing |*F*_calc_| with |*F*_obs_^detwinned^| (Fig. 2 ▸ *a*).

### 3.6. Optimization of the NCS averaging parameters   

The NCS rotation–translation operators and the shape of the averaging mask have to be accurately determined for effective use of NCS averaging to improve the twinning portions and phases. For the AiV1 crystals, the fivefold NCS operators were determined by aligning the icosahedral symmetry with the 23 cubic symmetry of the crystal (Figs. 1 ▸ *a*, 1 ▸ *b* and 1 ▸ *d* and Supplementary Fig. S2*b*). However, the initial averaging mask derived by including all voxels within 5 Å of any atom of the BEV model could have had an incorrect shape because of the differences in the capsids of BEV and AiV1. Therefore, a correlation map-based mask (Vellieux *et al.*, 1995 ▸) was prepared using the following steps. A correlation map was calculated with a voxel size of 4.5 Å. Each voxel of the correlation map corresponded to 265 voxels of the AiV1 electron-density map (voxel size 0.7 Å). Each voxel in the correlation map was assigned a value of the correlation coefficient calculated by comparing the corresponding 265 electron-density map values of voxels in the five NCS-related volumes. The correlation map was calculated using *COMA* (Kleywegt & Jones, 1999 ▸). A cutoff value of 0.65 was used for including the voxels from the correlation map into the correlation map-based mask. The use of the correlation map-based mask resulted in a decreased *R* factor comparing |*F*_calc_| and |*F*_obs_^detwinned^| relative to when the BEV-derived mask was used (Fig. 2 ▸ *a*). *Ex post*, we could also show that the use of the correlation map-based mask resulted in decreased phase differences from the phases derived from the final AiV1 model (Fig. 2 ▸ *b*). This indicated that the correlation map could be used to improve the shape of the mask used for NCS averaging, even for crystals affected by perfect hemihedral twinning.

### 3.7. Quality of electron-density maps   

The interpretability of an electron-density map calculated from the detwinned structure-factor amplitudes depends on the similarity of the phasing/detwinning model to the crystallized structure. Several maps with a varying utility for model building were obtained in the course of the AiV1 structure determination. The maps were closely inspected in terms of the presence of features corresponding to the AiV1 structure that were different from the molecular-replacement model. The quality of the phases used in map calculations was checked *ex post* by calculating phase-difference plots comparing the phases used to calculate maps at different stages of structure determination with phases from the final AiV1 model refined by ten cycles of NCS averaging (Fig. 2 ▸ *b*). The initial (2|*F*_obs_^detwinned^| − |*F*_calc_|), φ_calc_ map was calculated based on the phases and the twinning portions derived from the BEV model converted to a polyalanine chain. The map was strongly affected by model bias and did not show any features other than those of the BEV structure (Figs. 2 ▸ *b* and 3 ▸ *a*). The phases and the twinning portions were refined by ten cycles of NCS averaging (Figs. 2 ▸ *b* and 3 ▸ *b*). The resulting map was used to determine the correlation map-based mask. To utilize the availability of the numerous picornavirus models determined to atomic resolutions, a map was calculated by combining ten picornavirus models in order to calculate the initial phases and the twinning portions. Subsequently, the phases and the twinning portions were refined by 30 cycles of NCS averaging using the correlation map-based mask (Figs. 2 ▸ *b*, 3 ▸ *c* and 2 ▸ *d*). The resulting map was of sufficient quality to enable an initial manual model build. During the model building the electron-density maps were frequently recalculated with the initial phases derived from the latest model followed by 30 cycles of NCS averaging (Figs. 2 ▸ *b* and 3 ▸ *e*). The maps calculated using the phases and the twinning portions refined by NCS averaging exhibited clearer features than the map calculated with the phases and the twinning portions derived from the final AiV1 model (Figs. 2 ▸ *b* and 3 ▸ *f*).

### 3.8. Convergence radius of the detwinning procedure   

To test the limits of the convergence radius of the detwinning approach towards the correct phase solution, a molecular-replacement model with a different structure from that of the picornavirus capsid (bacteriophage φCb5; PDB entry 2w4y; Plevka *et al.*, 2009 ▸) was tested. A map calculated with the phases and the twinning portions derived from the φCb5 PDB model was affected by strong model bias and exhibited features of the φCb5 structure (Figs. 2 ▸ *b* and 3 ▸ *g*). After 30 cycles of NCS averaging, the electron-density map did not resemble φCb5; however, the map was uninterpretable (Figs. 2 ▸ *b* and 3 ▸ *h*). This indicated that the combination of molecular-replacement model-based detwinning with NCS averaging is a relatively safe approach for the removal of model bias because the calculation either converged to the correct structure (if the initial phasing model was sufficiently similar to the crystallized structure) or produced an uninterpretable map (if the initial model was too different from the crystallized structure). The *R* factors comparing |*F*_obs_^detwinned^| with |*F*_calc_| were similar for the φCb5 and BEV structures (Fig. 2 ▸ *a*). However, the NCS averaging produced a lower *R* factor in the phasing attempt initiated with the BEV model (Fig. 2 ▸ *a*). The quality of the phases obtained from the BEV and φCb5 models and the subsequent NCS averaging refinements was evaluated *ex post* by comparing the phases calculated from the final AiV1 model and refined by 30 cycles of NCS averaging (Fig. 2 ▸ *b*).

**Figure 4:**
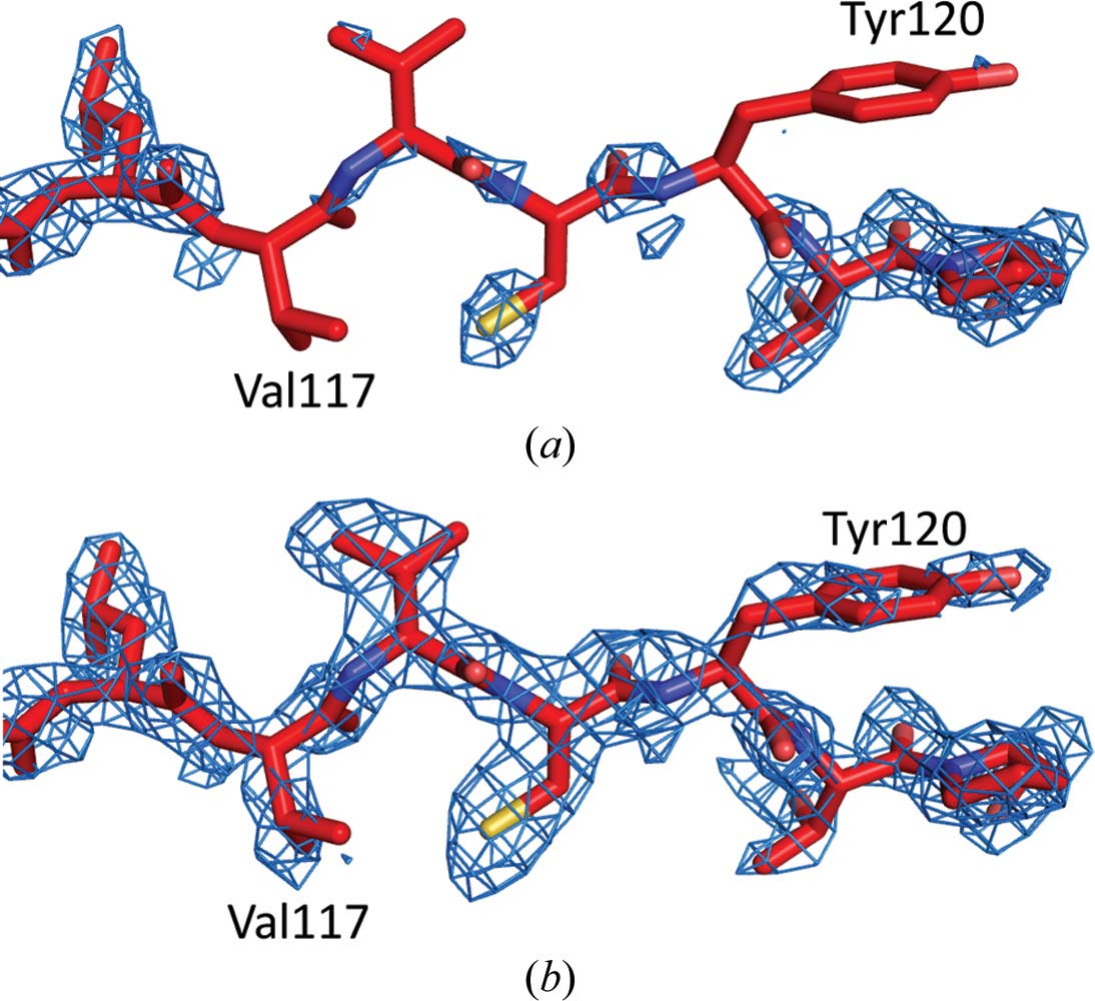
Electron density of a missing part of the structure can be recovered by a combination of the detwinning procedure and NCS averaging. (*a*) An OMIT (2|*F*_obs_^detwinned^| − |*F*_calc_|), φ_calc_ map calculated using the phases and the twinning portions derived from the final AiV1 model with deleted residues 117–120 of VP2. (*b*) An electron-density map for the missing part was recovered by ten cycles of NCS averaging.

The effectiveness of NCS averaging in obtaining the correct phases and the twinning portions was tested by calculating an OMIT map with the phases and the twinning portions derived from the final AiV1 model with residues 117–120 of VP2 deleted. The resulting OMIT map lacked the electron density corresponding to the deleted residues (Fig. 4 ▸ *a*). However, the electron density of the deleted residues could be recovered by ten cycles of NCS averaging (Fig. 4 ▸ *b*).

### 3.9. Size of the twin domains in comparison to the coherence length of the X-ray beam   

The crystallization conditions from which the twinned *I*23 crystal was obtained also produced crystals with apparent space group *P*4_2_32. The unit-cell size of the *P*4_2_32 crystal (*a* = 351.1 Å) was nearly identical to the unit cell of the *I*23 crystal (*a* = 350.8 Å). A statistical analysis of the reflection intensities from the *P*4_2_32 data set produced values that were even lower than the values expected for perfectly hemihedrally twinned data (Table 2 ▸). The native Patterson function calculated from the *P*4_2_32 data did not contain any large off-origin peaks. We interpret the statistics by proposing that the *P*4_2_32 crystal was built from the same domains as the *I*23 twinned crystals; however, in the *P*4_2_32 crystal the domains were smaller than the coherent length of the X-ray beam. Thus, the X-rays diffracted from the individual domains in the different orientations interacted as waves. The complex interaction of the diffracted X-rays might have resulted in the observed low twinning statistics (Table 2 ▸). This is in contrast to twinning, where the crystal domains are large relative to the coherence length and the diffracted beams sum their intensities. The *I*23 AiV1 unit cell contains one particle in the corner (fractional coordinates 0, 0, 0) and another particle with an identical orientation in the centre (0.5, 0.5, 0.5). The *P*4_2_32 unit cell might also contain two virus particles located at (0, 0, 0) and (0.5, 0.5, 0.5); however, the particle in the centre is rotated 90° relative to the particle in the corner (Supplementary Fig. S2*c*). The possibility of accommodating both of the 90° rotation-related particle orientations in the AiV1 crystal is consistent with the hemihedral twinning observed in the *I*23 crystal. The *P*4_2_32 data set did not produce an interpretable electron-density map even when it was phased using the final AiV1 model.

### 3.10. Model quality   

The electron-density map obtained after 30 cycles of real-space NCS averaging combined with detwinning was interpretable for most of the AiV1 structure. However, some regions, including the surface loops of VP1 located close to the icosahedral fivefold axes and the N-terminal arms of the capsid proteins located on the inside of the capsid, were difficult to interpret. The electron-density map of these parts became clearer when intermediate AiV1 models were used to calculate the initial phases in the detwinning procedure. For model construction, manual model building using *O* and *Coot* (Jones *et al.*, 1990 ▸; Emsley *et al.*, 2010 ▸) was alternated with refinement using *CNS* with the input files minimize_twin.inp and bindividual_twin.inp (Brunger, 2007 ▸).

The final AiV1 model includes residues 1–83 and 88–233 of VP1, residues 13–55, 64–75 and 112–370 of VP2 and residues 1–220 of VP3 together with 173 water molecules within one icosahedral asymmetric unit of the virion. The final crystallographic *R* factor (0.33) is high relative to the *R*_merge_ (0.166) of the 2.1 Å resolution data set (Table 3 ▸). The high *R* value might be owing to the complicated refinement using data affected by perfect hemihedral twinning.

The *R*_free_ factor (Brünger, 1992 ▸) was not calculated because it was not possible to select a set of reflections that would be independent of the reflections in the part of the data set used for the refinement (Fabiola *et al.*, 2006 ▸; Kleywegt & Brünger, 1996 ▸). To avoid correlations between working and free sets, the free-set reflections would need to be selected within fivefold NCS-related groups. In addition, both of the twin operator-related reflections would have to be included in the test set. However, it has been shown previously that it is not sufficient to select the *R*_free_ set in thin resolution shells because the reflections are correlated not only within the resolution shell but also with the neighbouring reflections of higher and lower resolution (Chen *et al.*, 1999 ▸). Thus, if calculated, *R*_free_ would be very similar to the *R* value owing to the fivefold NCS and the twin operator present in the diffraction data (Kleywegt & Brünger, 1996 ▸). Instead of using *R*_free_, the optimal weight of the X-ray refinement function relative to the energy minimization of the model was determined by checking the geometry of the model based on the r.m.s.d. of bond angles and lengths (Kleywegt, 2000 ▸).

### 3.11. Utility of the detwinning procedure for other perfectly hemihedrally twinned data sets   

The determination of a macromolecular structure from diffraction data affected by perfect hemihedral twinning is challenging because (i) the data cannot be detwinned unless a sufficiently similar model is available and (ii) even if a suitable model is available, the calculated electron-density map is affected by more extensive model bias than with untwinned data. However, here we show that it is possible to detwin perfectly hemihedrally twinned data and solve the structure in the presence of fivefold NCS. The best available molecular-replacement model (PDB entry 1cov) had 16% sequence identity and a 1.6 Å r.m.s.d. of C^α^ atoms for the 67% of the AiV1 residues that could be aligned (Table 4 ▸). The NCS averaging procedure reduced the model bias introduced by the differences between the molecular-replacement model and the crystallized structure. In the test case of bacteriophage φCb5, the procedure failed and produced an uninterpretable electron-density map. This functions as a safety check preventing the construction of structures biased towards the molecular-replacement model. The approach presented here could be used for other crystals affected by perfect hemihedral twinning that contain at least fivefold NCS.

## Supplementary Material

PDB reference: *Aichi virus 1*, 5aoo

Supplementary figures.. DOI: 10.1107/S2053230X16000923/hv5315sup1.pdf

## Figures and Tables

**Figure 3:**
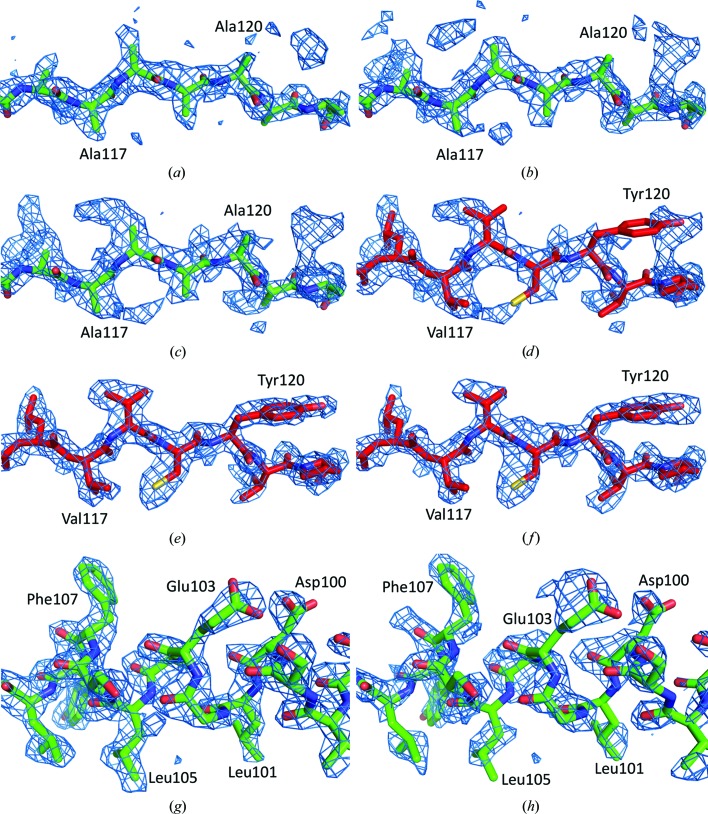
Comparison of (2|*F*_obs_^detwinned^| − |*F*_calc_|), φ_calc_ electron-density maps at various stages of AiV1 structure determination. (*a*) Electron-density map calculated using the phases and twinning portions derived from the BEV model converted to a polyalanine chain. The BEV model converted to polyalanine is shown in stick representation with the C atoms coloured green. (*b*) Electron-density map calculated using the phases and twinning portions from the BEV model converted to polyalanine and refined by ten cycles of NCS averaging using the BEV-derived mask. The BEV model converted to polyalanine is shown in stick representation with the C atoms coloured green. (*c*) Electron-density map calculated using the phases and twinning portions from the BEV model converted to polyalanine and refined by 30 cycles of NCS averaging using the correlation map-derived mask. See §[Sec sec3.6]3.6 for details of the mask preparation. The BEV model converted to polyalanine is shown in stick representation with the C atoms coloured green. (*d*) The same electron-density map as in (*c*) with the final AiV1 model shown in stick representation with the C atoms coloured red. (*e*) An electron-density map calculated using the phases and the twinning portions derived from the final AiV1 model. The final AiV1 model is shown in stick representation with the C atoms coloured red. (*f*) Electron-density map calculated using the phases and twinning portions derived from the final AiV1 structure and refined by ten cycles of NCS averaging. The final AiV1 model is shown in stick representation with the C atoms coloured red. (*g*) Electron-density map calculated using the phases and twinning portions derived from the φCb5 structure. The model of φCb5 is shown in stick representation with the C atoms coloured green. (*h*) Electron-density map calculated using the phases and twinning portions from the φCb5 structure and refined by ten cycles of NCS averaging. The model of φCb5 is shown in stick representation with the C atoms coloured green.

**Table 1:** Analysis of rotational symmetry in AiV1 *I*23 diffraction data

Symmetry	Symmetry-axis direction	Lattice transformation	*R*_meas_ †	Correlation coefficient	No. of compared values
Identity			0.080	0.99	10724
Twofold *l*	(0 0 1)	(−*h*, −*k*, *l*)	0.058	0.99	143
Twofold *k*	(0 1 0)	(−*h*, *k*, −*l*)	0.077	0.99	7282
Twofold *h*	(1 0 0)	(*h*, −*k*, −*l*)	0.081	0.99	17996
Twofold	(1 −1 0)	(−*k*, −*h*, −*l*)	0.093	0.98	33900
Twofold	(0 1 −1)	(−*h*, −*l*, −*k*)	0.088	0.98	41117
Twofold	(1 0 −1)	(−*l*, −*k*, −*h*)	0.098	0.98	20700
Twofold	(1 1 0)	(*k*, *h*, −*l*)	0.084	0.99	2570
Twofold	(1 0 1)	(*l*, −*k*, *h*)	0.099	0.98	11137
Twofold	(0 1 1)	(−*h*, *l*, *k*)	0.087	0.98	15869
Threefold	(1 −1 −1)	(−*k*, *l*, −*h*), (−*l*, −*h*, *k*)	0.086	0.98	9197
Threefold	(1 1 −1)	(−*l*, *h*, −*k*), (*k*, −*l*, −*h*)	0.089	0.98	25697
Threefold	(1 −1 1)	(*l*, −*h*, −*k*), (−*k*, −*l*, *h*)	0.084	0.98	8964
Threefold	(1 1 1)	(*k*, *l*, *h*), (*l*, *h*, *k*)	0.087	0.98	52513
Fourfold *l*	(0 0 1)	(−*k*, *h*, *l*), (*k*, −*h*, *l*)	0.094	0.98	24095
Fourfold *k*	(0 1 0)	(*l*, *k*, −*h*), (−*l*, *k*, *h*)	0.105	0.98	7369
Fourfold *h*	(1 0 0)	(*h*, *l*, −*k*), (*h*, −*l*, *k*)	0.087	0.98	1793

† *R*_meas_ = 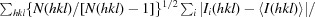 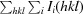.

**Table 2:** Twinning statistics for the AiV1 diffraction data

	Twinning test						
		Mean acentric moments of *I* (moment No.)			Mean centric moments of *I* (moment No.)		
	*L* test	2	3	4	2	3	4
Untwinned expected value	0.50	2.0	6.0	24.0	3.0	15.0	105.0
Twinned expected value	0.38	1.5	3.0	7.5	2.0	6.0	24.0
*I*23 data (72.0–2.3 Å)	0.46	2.1	6.2	25.4	2.8	11.8	71.3
*P*4_2_32 data (68.0–3.5 Å)	0.23	1.2	1.7	2.7	1.4	1.9	4.4

**Table 3:** Diffraction data and structure-quality indicators Values in parentheses are for the highest resolution shell. Because of the perfect hemihedral twinning, the data were integrated and scaled in space group *I*432. A greater than 90% complete data set with a resolution of 2.1 Å was obtained from the first 160 images (0.1° oscillation per frame) that were least affected by radiation damage. For refinement, the data were expanded to space group *I*23.

	Twinned crystal processed in space group			Primitive cell
	*I*23	*I*432	*I*23 expanded from *I*432	*P*4_2_32
Unit-cell parameter *a* (Å)	350.8	350.8		351.1
Resolution	72.0–2.3 (2.34–2.30)	72.0–2.1 (2.14–2.10)		68.0–3.5 (3.56–3.50)
No. of observations	902384 (43198)	719342 (21777)		247619 (12984)
No. of unique observations	282853 (14471)	186646 (8521)	367359 (16771)	86648 (4550)
Observation multiplicity	3.2 (3.0)	3.9 (2.6)		2.9 (2.9)
Completeness (%)	91.0 (94.0)	90.0 (83.6)		93.6 (94.1)
*R*_merge_ †	0.154 (0.628)	0.166 (0.872)		0.252 (1.04)
〈*I*/σ(*I*)〉	6.0 (1.8)	5.5 (1.1)		4.5 (0.9)
*R* factor‡			0.33	
No. of protein atoms§			5791	
No. of water atoms§			147	
Average *B* factor (Å^2^)			22.9	
Ramachandran statistics¶				
Preferred regions (%)			95.7	
Allowed regions (%)			4.0	
Disallowed regions (%)			0.3	
R.m.s.d., bond angles (°)			1.43	
R.m.s.d., bond lengths (Å)			0.013	

† *R*_merge_ = 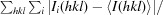 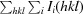.

‡ The value of the crystallographic *R* factor is relatively high when compared with the *R*_merge_ of the 2.1 Å resolution data set. The high value might be owing to the complicated refinement when using the twinned data and/or because the crystal might have been also affected by defects other than the perfect hemihedral twinning.

§ Data are given for one icosahedral asymmetric unit.

¶ According to *MolProbity* (Chen *et al.*, 2010 ▸).

**Table 4:** Comparison of molecular-replacement models The icosahedral asymmetric units were used as rigid bodies in all cases. *O* (Jones *et al.*, 1990 ▸) was used for superposition of the molecules. The cutoff for the inclusion of residues in the r.m.s.d calculation was 3.8 Å

MR model (PDB code)	1bev	1cov	1ev1	1hxs	1tmf	2mev	2wff	2x5i	3vbf	4iv1
R.m.s.d. from final AiV1 structure† (Å)	1.64	1.64	1.64	1.60	1.63	1.53	1.76	1.62	1.57	1.72
Fraction of aligned residues‡ (%)	70	67	65	64	69	69	79	66	65	79
Sequence identity to AiV1 (%)	16	16	15	16	19	19	20	15	17	18
*R* factor after MR	0.509	0.508	0.512	0.514	0.514	0.511	0.515	0.515	0.514	0.519

† R.m.s. deviations of superimposed C^α^ atoms of the respective structures from the final AiV1 model.

‡ Percentage of available amino-acid residues used for the calculations.
