# Supplementary material for: The use of noncrystallographic symmetry averaging to solve structures from data affected by perfect hemihedral twinning
Source: Acta Crystallogr F Struct Biol Commun. 2016 Feb 16;72(Pt 3):188–97. doi: 10.1107/S2053230X16000923 (PMC4774877; doi:10.1107/S2053230X16000923)
Supplement: Supplementary file 1 [file f-72-00188-sup1.pdf]

STRUCTURAL BIOLOGY
COMMUNICATIONS

Volume 72 (2016)

Supporting information for article:

The use of noncrystallographic symmetry averaging to solve structures from data affected by perfect hemihedral twinning

Charles Sabin and Pavel Plevka

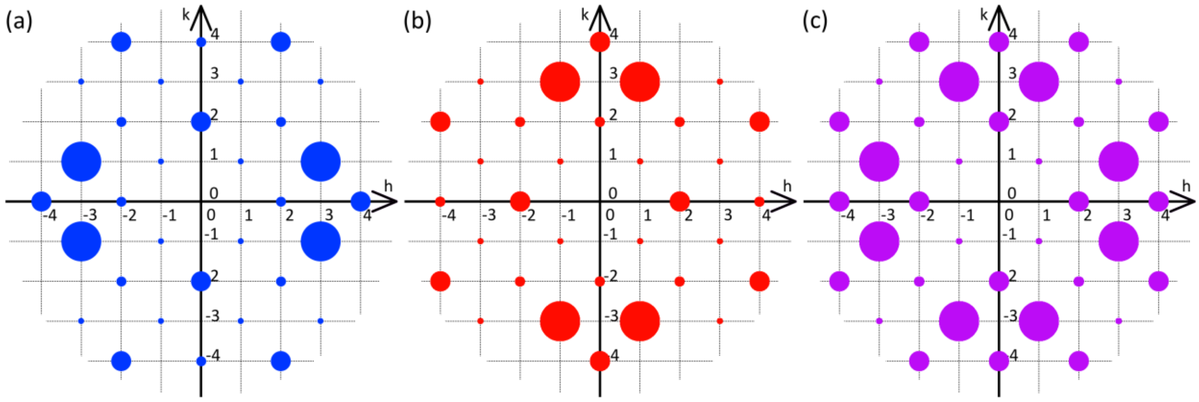

Figure S1 Effect of perfect hemihedral twinning on symmetry of I23 cubic diffraction pattern. The images show 0kl planes of reflections with twofold symmetry axes along k and l for the 0° (a) and 90° (b) rotated twin variants. The sum of the two patterns corresponding to the perfect hemihedral twin is shown in (c). Please note that the summed pattern in (c) has fourfold symmetry.

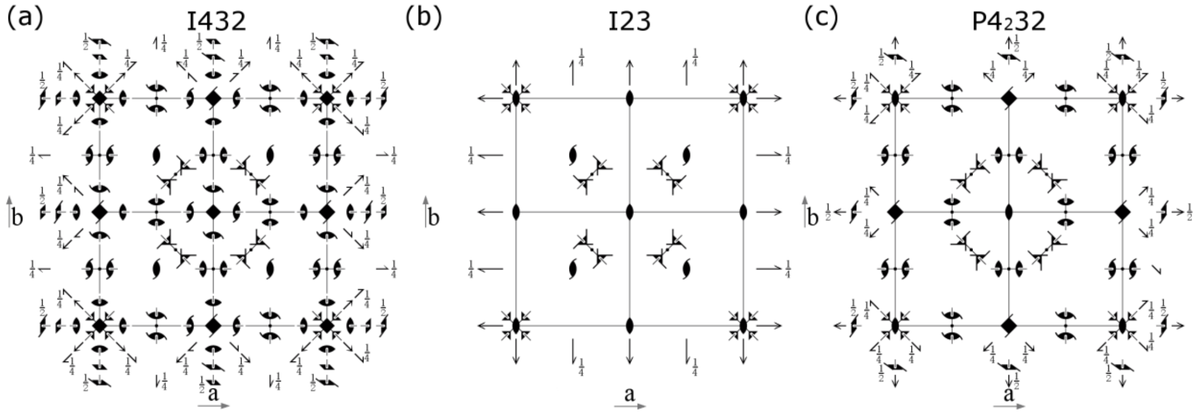

Figure S2 I23 (a), I432 (b), and P$4_2$32 (c) space group symmetry diagrams.
